# Renal biomarkers in cats: A review of the current status in chronic kidney disease

**DOI:** 10.1111/jvim.16377

**Published:** 2022-02-26

**Authors:** Thirawut Kongtasai, Dominique Paepe, Evelyne Meyer, Femke Mortier, Sofie Marynissen, Lisa Stammeleer, Pieter Defauw, Sylvie Daminet

**Affiliations:** ^1^ Small Animal Department, Faculty of Veterinary Science Ghent University Merelbeke Belgium; ^2^ Department of Clinical Sciences and Public Health, Faculty of Veterinary Science Mahidol University Nakhon Pathom Thailand; ^3^ Department of Pharmacology, Toxicology and Biochemistry, Faculty of Veterinary Medicine Ghent University Merelbeke Belgium; ^4^ Lumbry Park Veterinary Specialists Alton United Kingdom

**Keywords:** feline, fibroblast growth factor‐23, kidney injury molecule‐1, liver‐type fatty acid‐binding protein, neutrophil gelatinase‐associated lipocalin, transforming growth factor‐β1

## Abstract

Serum creatinine concentration, the classical biomarker of chronic kidney disease (CKD) in cats, has important limitations that decrease its value as a biomarker of early CKD. Recently, serum symmetric dimethylarginine concentration was introduced as a novel glomerular filtration rate biomarker for the early detection of CKD in cats. However, data on its specificity are still limited. The limitations of conventional biomarkers and the desire for early therapeutic intervention in cats with CKD to improve outcomes have prompted the discovery and validation of novel renal biomarkers to detect glomerular or tubular dysfunction. Changes in the serum or urinary concentrations of these biomarkers may indicate early kidney damage or predict the progression of kidney before changes in conventional biomarkers are detectable. This review summarizes current knowledge on renal biomarkers in CKD in cats, a field that has progressed substantially over the last 5 years.

**ABBREVIATIONS**
AKI: acute kidney injury
ALB: albumin
CKD: chronic kidney disease
CysB: cystatin B
CysC: cystatin C
FGF‐23: fibroblast growth factor‐23
GFR: glomerular filtration rate
HMW: high molecular weight
HSP‐72: heat shock protein‐72
IMW: intermediate molecular weight
kDa: kilodalton
KIM‐1: kidney injury molecule‐1
L‐FABP: liver‐type fatty acid‐binding protein
LMW: low molecular weight
MMP: matrix metalloproteinase
NAG: N‐acetyl‐b‐d‐glucosaminidase
NGAL: neutrophil gelatinase‐associated lipocalin
p: plasma
PENIA: particle‐enhanced nephelometric immunoassay
PETIA: particle‐enhanced turbidimetric immunoassay
PIIINP: procollagen type III amino‐terminal propeptide
PTH: parathyroid hormone
RBP: retinal‐binding proteins
s: serum
sCr: Serum creatinine
SDMA: symmetric dimethylarginine
SDS‐PAGE: sodium dodecyl sulfate‐polyacrylamide gel electrophoresis
TGF‐β1: transforming growth factor‐β1
u: urinary
UPC: urinary protein : creatinine ratio
USG: urine specific gravity

## 1 INTRODUCTION

Chronic kidney disease (CKD) is a common diagnosis in older cats. The overall prevalence of CKD in cats is approximately 2% to 4%,^1^, ^2^ and increases to 30% to 40% in cats >10 years of age.^3^, ^4^ Data from 3 million dogs also support that the prevalence of CKD increases with age.^5^ In cats, several factors contribute to kidney injury are implicated in CKD,^6^ and renal damage tends to progress over time at an unpredictable rate.^6^ Tubulointerstitial inflammation is the most common histopathological feature of CKD in cats, with fibrotic changes signifying a worse prognosis.^7^, ^8^

Clinically, CKD in cats usually is diagnosed at a late stage based on a combination of compatible clinical signs, azotemia, and an inappropriate urine specific gravity (USG).^9^, ^10^ Although a USG <1.035 is regarded as abnormal in cats with dehydration or azotemia, urinary concentrating ability is occasionally intact in cats with CKD.^9^, ^11^ Furthermore, inappropriately dilute urine also can be non‐renal in origin, for instance secondary to certain drugs (eg, diuretics), glucosuria, electrolyte imbalances, or liver disease.^12^

Measurement of glomerular filtration rate (GFR) is the gold standard and most sensitive test for impaired renal function.^13^ The GFR can be determined directly by measuring the clearance of an endogenous or exogenous filtration marker.^13^ Although limited sampling techniques have been developed to improve the practical use of GFR measurement in cats, none of these current techniques is feasible for routine veterinary practice, and direct GFR measurement is still mostly a research tool.^14^, ^15^, ^16^, ^17^

Proteinuria as assessed by the urinary protein : creatinine ratio (UPC) is a routine renal biomarker and traditional hallmark of glomerular disease but also increases with tubular dysfunction in cats.^8^, ^18^, ^19^ Persistent renal proteinuria without azotemia may indicate early glomerular disease in cats.^9^ Epidemiologic studies have suggested that UPC is prognostic for survival, progression and the development of CKD in cats.^3^, ^20^, ^21^, ^22^ Results of the UPC values in cats commonly are influenced by non‐renal diseases such as hyperthyroidism, viral infections, and lower urinary tract diseases, which substantially decrease the specificity of UPC for kidney disease.^6^, ^23^, ^24^

To delay CKD progression, treatment ideally should be instituted as soon as the diagnosis is achieved.^25^ The search for serum or urine renal biomarkers that are more sensitive for detecting early kidney damage or small decreases in kidney function is driven by the limitations and poor sensitivities of traditional biomarkers such as serum creatinine (sCr) concentration.^26^

The innovative concept that acute kidney injury (AKI) and CKD in cats and dogs are interconnected processes recently has been proposed, analogous to the situation in humans. This concept states that sustained or serious kidney injury, such as that occurring during an episode of AKI, could lead to the development of CKD and vice versa. This ongoing kidney injury generally occurs before a noticeable decrease in GFR.^6^, ^27^ Furthermore, “acute kidney stress” was introduced as a concept in humans and dogs to describe a predisposition to AKI or a very early insult to the kidney before AKI.^28^ Combining these concepts, it can be hypothesized that kidney stress or early kidney injury may be associated with the development and progression of CKD. Therefore, renal biomarkers that can identify active kidney stress or injury have the potential to detect CKD earlier than biomarkers that are surrogates of decreased GFR. Moreover, these injury biomarkers, especially those in urine, can also specifically localize injury to glomeruli or tubules. Furthermore, these biomarkers might have the potential to predict the development of CKD, monitor recovery, and facilitate prognostication.^6^, ^29^

In this review, we categorize and discuss renal biomarkers measured in feline blood and urine based on their origin and role in kidney disease. In particular, we describe biomarkers of GFR, metabolic derangement, glomerular vs tubular injury or dysfunction, and renal fibrosis, focusing on biomarkers described using new data obtained the last 5 years.

## 2 CURRENT STATUS OF CLINICAL DIAGNOSIS OF CKD

### 2.1 Systemic biomarkers of GFR

The serum or plasma biomarkers under investigation in cats with CKD are summarized in Table 1.

**TABLE 1:** Overview of systemic renal biomarkers in cats with CKD

Biomarker	Origin	Type of molecule	Mechanism underlying increased levels	Validated assays in cats	Changes in cats with azotemic‐CKD	Non‐renal factors to consider
Biomarkers of GFR						
SDMA	All nucleated cells	Methylated amino acid	Decreased GFR	Liquid chromatography‐mass spectroscopy^30^	Increased^30^	
CysC	All nucleated cells	LMW protein and cysteine protease inhibitor	Decreased GFR	Human PENIA^31^ Human PETIA^32^, ^33^	Increased^31^, ^32^, ^34^, ^35^ or insignificant^36^	Hyperthyroidism^24^, ^36^
Biomarker of metabolic derangement						
FGF‐23	Osteoblasts and osteocytes	Phosphaturic hormone	Altered phosphate metabolism	ELISA^37^	Increased^37^, ^38^	Hyperthyroidism^39^

Abbreviations: CysC, cystatin C; FGF‐23, fibroblast growth factor‐23; GFR, glomerular filtration rate; LMW, low molecular weight; PENIA, particle‐enhanced nephelometric immunoassay; PETIA, particle‐enhanced turbidimetric immunoassay; SDMA, symmetric dimethylarginine.

Because of logistical drawbacks, GFR is usually indirectly estimated by measuring serum biomarker concentrations. The traditional GFR biomarker is sCr, which is widely used to diagnose CKD because it is economical, readily available, easy to measure, and shows low intra‐individual variability.^9^, ^40^ Also when within reference range, serial monitoring of sCr can be used to detect early change in renal function.^10^, ^40^ However, sCr has important limitations. First, it is influenced by non‐renal factors, especially muscle mass.^30^, ^41^, ^42^ Second, sCr has a low sensitivity to detect an early decrease in GFR and cannot detect kidney damage that does not affect GFR.^6^, ^29^ Third, its wide reference interval because of high inter‐individual variability and its analytical variability can lead to misinterpretation in clinical practice.^43^, ^44^ These major limitations hamper the utility of sCr to detect early CKD in cats, resulting in a search for other indirect GFR biomarkers such as symmetric dimethylarginine (SDMA).

Besides the conventional functional biomarker, sCr, which is the main representative of this group, SDMA concentration recently has been described as a novel GFR biomarker for the diagnosis of CKD in cats.^45^, ^46^ Its main advantage is that it is more sensitive than sCr concentration because it can detect a smaller decrease in GFR, increasing when the GFR decreases by approximately 40% in cats.^25^, ^30^ Another advantage is that SDMA concentration is not affected by muscle mass.^47^, ^48^, ^49^ However, breed‐related differences in SDMA concentration have been noted and require further exploration.^48^ A more important limitation of SDMA concentration is that any increase must be interpreted with caution because data on its specificity are scarce.^46^, ^50^ International Renal Interest Society (IRIS) has recently incorporated SDMA concentration into guidelines for CKD staging.^51^ According to IRIS, persistently increased SDMA concentration may indicate early CKD (IRIS stage 1).^51^ Hence, multiple blood samplings are required when interpreting SDMA concentration. Because extensive reviews on the current knowledge of SDMA in dogs and cats have been published recently,^29^, ^46^, ^52^, ^53^ SDMA will not be discussed in depth in the present review. To date, the only other surrogate biomarker of GFR that has received attention in cats is serum cystatin C (sCysC). This marker will be reviewed below, but despite extensive analytical validation sCysC has failed to show clinical utility as a marker of GFR.^32^, ^36^

#### 2.1.1 Serum cystatin C

Cystatin C is a 13 kilodalton (kDa) non‐glycosylated protein produced by all nucleated cells at a constant rate. It is involved in intracellular protein catabolism as a proteinase inhibitor.^54^ Circulating CysC generally passes through the glomerular barrier without restriction.^55^ In the proximal tubules, CysC is reabsorbed via megalin receptors and then is metabolized completely.^56^ There is no proof that CysC is secreted by tubular cells into urine.^57^ In humans and dogs, sCysC concentration is used as GFR biomarker and is highly correlated with sCr and GFR.^34^, ^57^, ^58^, ^59^, ^60^ Also, a GFR equation based on the combination of sCysC and sCr has been shown to be the optimal equation to estimate GFR in humans.^61^

No cat‐specific assay is available for CysC measurement. Although particle‐enhanced nephelometric (PENIA) and particle‐enhanced turbidimetric (PETIA) sCysC immunoassays used in humans had acceptable precision and reproducibility in cats,^31^, ^32^, ^33^ the cross‐reactivity between the anti‐human CysC antibody and feline CysC was relatively low based on Western blot analysis.^31^, ^32^ This questions the reliability of the PETIA and PENIA used in humans for measurement of sCysC in cats. Moreover, sCysC concentrations significantly decrease after storage for only 5 months at both −20°C and −72°C.^62^

Age, sex, breed, and body weight do not affect sCysC concentrations in cats,^35^, ^63^ in contrast to humans and dogs.^64^, ^65^ No significant differences were found between pre‐ and post‐prandial sCysC concentrations in cats.^62^ Hyperthyroidism increases sCysC concentrations in cats independent of decreased renal function.^24^, ^36^ However, feline immunodeficiency virus infection has no effect on sCysC concentrations.^24^

Although initial studies indicated that CKD cats had significantly higher sCysC concentrations than healthy cats, there was overlap between groups.^31^, ^35^, ^58^ Also, despite a significant negative correlation with GFR, sCysC concentrations were not different for different IRIS stages within the group of CKD cats.^35^ The sensitivity of sCysC for the detection of decreased GFR was only 22%, and, in recent studies, sCysC concentrations could not differentiate CKD cats and healthy cats.^32^, ^36^ The correlation between GFR and sCysC was significantly weaker than that between GFR and sCr.^32^ Additionally, sCysC could not predict the development of azotemia after treatment of hyperthyroid cats.^36^ Overall, despite promising data in humans, sCysC is not a useful biomarker for renal function in cats.

## 3 BIOMARKERS OF METABOLIC DERANGEMENT

The decrease in phosphate excretion from diseased kidneys results in disturbances in the systemic calcium‐phosphate homeostasis characterized by increasing serum phosphate concentrations, stimulation of parathyroid hormone (PTH) secretion, and osteodystrophy. This systemic condition of calcium‐phosphate homeostasis disturbance caused by CKD is called chronic kidney disease‐mineral and bone disorder (CKD‐MBD).^66^ Recently, knowledge of this metabolic derangement in cats with CKD has been improved by studying how the phosphaturic hormone, fibroblast growth factor‐23 (FGF‐23), behaves in CKD cats and geriatric cats (Table 1).

### 3.1 Fibroblast growth factor‐23

Fibroblast growth factor‐23 is a hormone responsible for the regulation of phosphorus and calcitriol produced by osteocytes and osteoblasts.^67^, ^68^ Both hyperphosphatemia and increased blood calcitriol (active vitamin D) concentrations stimulate FGF‐23 production, which promotes urinary phosphorus excretion and decreases intestinal phosphorus reabsorption. Urinary phosphorus excretion is increased by the inhibition of proximal tubular sodium‐phosphorus cotransporters, whereas intestinal phosphorus reabsorption is decreased by impaired renal calcitriol production.^69^, ^70^, ^71^ The concentration of FGF‐23 also has been investigated as an early biomarker of CKD‐MBD in humans.^72^, ^73^, ^74^ An increase in FGF‐23 concentration is an early abnormality detected in people with early CKD, preceding increased serum phosphate and PTH concentrations.^74^, ^75^ Also, FGF‐23 was negatively correlated with estimated GFR (eGFR) in human patients.^76^

Different FGF‐23 ELISAs have been used in studies of cats. Initially, a commercial FGF‐23 sandwich ELISA designed for humans was validated to measure plasma FGF‐23 concentration in cats, with acceptable reproducibility, precision, and specificity.^37^ Plasma (p)FGF‐23 concentrations were stable for at least 7 days at 22°C and for at least 14 days at −20°C, and 4 freeze‐thaw cycles did not influence its concentration.^37^ Recently, a commercial ELISA designed to detect FGF‐23 in cats has been examined.^38^

Cats with CKD and hyperphosphatemia have higher pFGF‐23 concentrations than those with normophosphatemia within the same IRIS stage.^37^ In contrast, pFGF‐23 concentration is not correlated with phosphate concentration in cats with normal renal function.^77^, ^78^ It is still unclear whether this difference is specifically related to serum phosphorus concentration. Many other factors (eg, total calcium, magnesium, PTH, and PCV) also are associated with pFGF‐23 concentrations in CKD cats.^37^, ^79^ Hyperthyroidism seems to decrease pFGF‐23 concentrations in non‐azotemic cats, but its concentration cannot predict the development of azotemia after treatment.^39^ Feeding a renal diet decreased pFGF‐23 concentrations in both hyper‐ and normo‐phosphatemic CKD cats.^80^ Along with decreased pFGF‐23, significant changes in plasma phosphate and PTH concentrations also were found in the hyperphosphatemic group but not in the normophosphatemic group.^80^ Thus, lower pFGF‐23 concentrations may signify improvement of phosphate derangement in CKD cats.

Several studies have suggested that FGF‐23 might be a promising early biomarker for phosphate derangement in CKD in cats.^37^, ^38^, ^77^, ^78^, ^80^, ^81^ Despite similar phosphate, PTH, and sCr concentrations, baseline pFGF‐23 concentrations were significantly higher in non‐azotemic geriatric cats that developed azotemia within 12 months than in cats with stable renal function.^77^ Furthermore, pFGF‐23 was increased in non‐azotemic cats with SDMA concentrations >14 μg/dL despite an absence of hyperphosphatemia.^78^ These findings indicate that FGF‐23 might have diagnostic potential for early detection of CKD and early phosphate derangement in cats and that phosphate dysregulation may be ongoing in the early stage of CKD before azotemia and hyperphosphatemia occur. Plasma FGF‐23 concentrations were also significantly higher in cats with azotemic CKD than in healthy cats, and these concentrations significantly increased with the severity of CKD.^37^, ^38^ Also, pFGF‐23 concentration was identified as independent predictor of CKD progression (>25% increase in sCr) within 12 months of diagnosis of CKD on a population basis.^79^, ^81^ However, the studies described are population‐based studies and overlap identified between groups indicates that it is difficult to extrapolate to an individual cat basis.^79^, ^81^ In these studies, overlap existed in pFGF‐23 concentrations between the different study groups despite significantly different pFGF‐23 concentrations between groups.^37^, ^38^, ^77^, ^78^, ^80^, ^81^

Overall, FGF‐23 is an earlier biomarker for phosphate derangement as compared to other routine biomarkers such as PTH and phosphate, and could identify CKD cats benefiting from dietary management in an earlier stage. Additional studies are needed to define the true diagnostic potential of pFGF‐23 concentration as marker of early phosphate derangement or marker of CKD progression in the individual cat presented in clinical practice.

## 4 KIDNEY INJURY BIOMARKERS IN CATS WITH CKD

Biomarkers of glomerular and tubular injury or dysfunction primarily are detected in urine. Glomerular biomarkers are either intermediate (IMW) or high molecular weight (HMW) proteins, the concentrations of which increase in urine when the glomerular barrier becomes more permeable because of glomerular damage.^29^ Generally, most tubular biomarkers are LMW proteins, the urinary concentrations of which are low or undetectable when tubular function is normal. Tubular injury or dysfunction can cause defects in the resorptive capacity for these LMW proteins. Additionally, tubular cells can secrete some LMW proteins in response to direct tubular damage.^6^, ^29^ Tubulointerstitial nephritis commonly is found in CKD cats.^8^ This explains why CKD cats usually have mild proteinuria, and mostly changes in tubular biomarkers are expected. On the other hand, marked proteinuria or markedly increased glomerular biomarkers are expected in primary glomerular disease, which is uncommon in cats, except for renal amyloidosis in predisposed cat breeds.^8^, ^82^, ^83^

**FIGURE 1:**
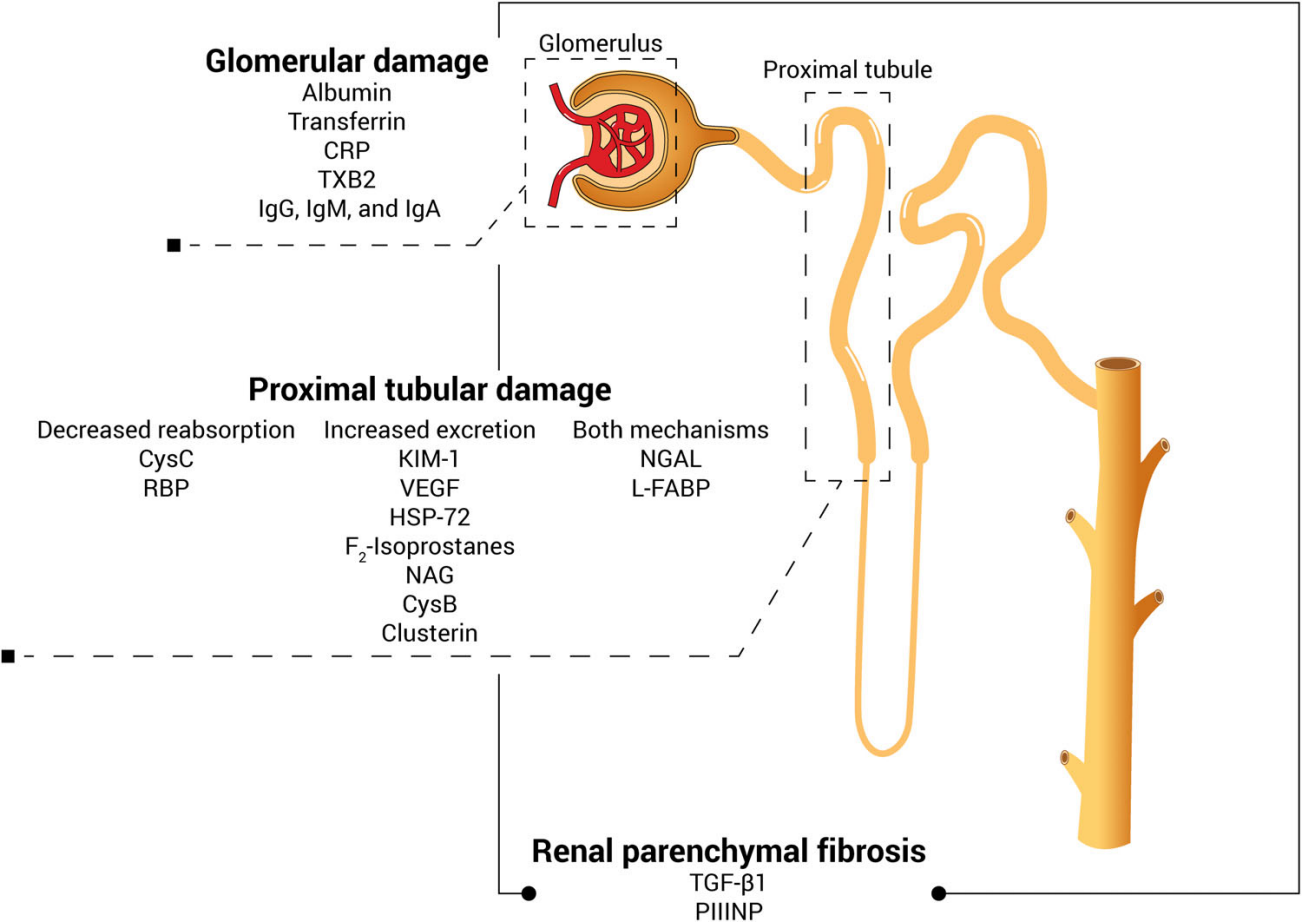
Overview of the urinary biomarkers in CKD in cats based on their origin. CRP, C‐reactive protein; CysB, cystatin B; CysC, cystatin C; HSP‐72, heat shock protein‐72; IgA, immunoglobulin A; IgG, immunoglobulin G; IgM, immunoglobulin M; KIM‐1, kidney injury molecule‐1; L‐FABP, liver‐type fatty acid‐binding protein; NAG, N‐acetyl‐b‐d‐glucosaminidase; NGAL, neutrophil gelatinase‐associated lipocalin; PIIINP, procollagen type III amino‐terminal propeptide; RBP, retinol‐binding protein; TGF‐β1, transforming growth factor‐β1; TXB2, thromboxane B2; VEGF, vascular endothelial growth factor

Other biomarkers also can be present in urine as a consequence of fibrosis or oxidative injury, or both reflecting the pathogenesis of CKD.^84^ Urinary biomarker concentrations usually are corrected by determining their ratio to urinary creatinine concentrations to adjust for variation in urinary volume and concentration.^85^ An overview of urinary renal biomarkers evaluated in CKD in cats based on their origin in the kidney is presented in Figure 1. Knowledge about urinary renal biomarkers in cats is summarized in Table 2.

**TABLE 2:** Overview of urinary renal biomarkers in cats with CKD

Biomarker	Origin	Type of molecule	Mechanism underlying increased levels	Validated assays in cats	Changes in cats with azotemic‐CKD	Non‐renal factors to consider
Biomarkers of glomerular damage						
Albumin	Hepatocytes	IMW protein	Increased glomerular permeability	Feline‐specific ELISA^20^ Human PETIA^33^ Electrophoresis^86^	Insignificant^33^	Lower urinary tract disease; systemic disease^86^, ^87^, ^88^, ^89^
Transferrin	Liver (primarily) and other tissues	IMW protein iron‐transporting protein	Increased glomerular permeability	SDS‐PAGE^90^	Increased^90^	
Biomarkers of tubular impairment						
L‐FABP	Proximal tubular cells	LMW and fatty acid‐binding proteins	Increased tubular excretion	ELISA^91^	Increased^91^, ^92^, ^93^	Hyperthyroidism^91^
NGAL	Epithelial cells of proximal tubules, neutrophils and other tissues	LMW protein and glycoprotein	Decreased tubular reabsorption and increased tubular excretion	ELISA^91^, ^94^	Increased^94^, ^95^ or insignificant^91^	Pyuria; systemic disease^95^
KIM‐1	Proximal tubular cells	Renal tubular transmembrane glycoprotein	Decreased reabsorption and increased excretion	ELISA^96^	Not assessed	
VEGF	Renal proximal tubules	Signaling protein	Decreased production	ELISA^97^	Decreased^97^	Hyperthyroidism^98^
CysC	All nucleated cells	LMW protein and cysteine protease inhibitor	Decreased tubular reabsorption	Human PENIA^31^ Human PETIA^32^, ^36^	Increased^32^ or decreased^36^	Hyperthyroidism^24^
HSP72	Renal tubular cells	Stress‐induced cytoprotective protein	Increased tubular excretion	ELISA^99^	Increased^99^	Urethral obstruction^99^
F_2_‐Isoprostanes	Kidney	Antioxidant	Increased production	ELISA^100^	Decreased^100^	Hyperthyroidism^101^
CysB	Proximal tubular cells	LMW protein and cysteine protease inhibitor	Ruptured and death of tubular epithelial cells	ELISA^27^	Not assessed	
Clusterin	Renal tubular cells	LMW protein and glycoprotein	Increased production	ELISA^27^	Not assessed	
Biomarkers of renal fibrosis						
TGF‐β1	Parenchymal and inflammatory cells	Cytokine and pro‐fibrotic mediator	Increased production	ELISA^102^, ^103^	Increased^102^ or insignificat^103^	
PIIINP	Collagen	Amino‐terminal propeptide of type III collagen	Decreased reabsorption	ELISA^104^	Increased^104^	

Abbreviations: CysB cystatin B, CysC, cystatin C; HSP‐72, heat shock protein‐72; IMW, intermediate molecular weight; KIM‐1, kidney injury molecule‐1; L‐FABP, liver‐type fatty acid‐binding protein; LMW, low molecular weight; NGAL, neutrophil gelatinase‐associated lipocalin; PENIA, particle‐enhanced nephelometric immunoassay.

Other glomerular biomarkers such as immunoglobulins G, M, and A as well as C‐reactive protein and thromboxane B2 have been studied in dogs, but have not yet been investigated in cats.^29^, ^105^ Also, no recent information of other tubular biomarkers previously investigated in cats, namely urinary N‐acetyl‐b‐d‐glucosaminidase (NAG) and retinal‐binding proteins (RBP) have been published since previous reviews on this topic.^29^, ^105^ Therefore, these biomarkers will not be discussed in the present review.

### 4.1 Biomarkers of glomerular injury

#### 4.1.1 Albumin

Albumin (ALB) is an IMW (65 kDa) protein produced by hepatocytes. In general, circulating ALB cannot freely pass through an intact glomerular barrier because its size exceeds the glomerular permeability threshold. Small quantities of ALB that pass through the glomerulus into tubular fluid are completely reabsorbed by proximal tubular cells. Consequently, healthy cats have <1 mg/dL of ALB in their urine (uALB). Thus, both glomerular and tubular dysfunction can result in albuminuria.^106^ Microalbuminuria and overt albuminuria are defined as uALB concentrations between 1 to30 mg/dL and >30 mg/dL, respectively.^106^, ^107^ Albuminuria and increased uALB/Cr have been evaluated in AKI and CKD in dogs,^87^, ^108^, ^109^ and microalbuminuria was associated with increased risk of death in dogs with critical conditions.^87^

In the past, semi‐quantitative techniques including urine dipstick colorimetric testing and sulfosalicylic acid turbidity testing were used as screening tools for albuminuria in cats. However, their poor specificity for the detection of albuminuria in cats decreases the utility of these tests.^86^, ^110^, ^111^ In recent years, more accurate techniques have been validated to quantify uALB concentration in cats, including a feline‐specific sandwich ELISA, a PETIA used in humans, and electrophoresis, all with acceptable precision and reproducibility.^20^, ^33^, ^86^, ^90^ However, the availability of these tests to general veterinary practitioners remains limited.

Several studies have demonstrated the diagnostic and prognostic value of uALB concentration for CKD in cats. Correlations between albuminuria and proteinuria were reported to be significant.^20^, ^23^, ^33^, ^110^ Similar to proteinuria, albuminuria was negatively associated with survival in a population of client‐owned cats with or without CKD.^20^ However, guidelines to predict survival in an individual cat based on severity of albuminuria currently do not exist. Cats with CKD and higher uALB/Cr ratios had shorter survival times than those with lower uALB/Cr ratios.^20^ Urinary ALB/Cr ratios in cats with stage 1 CKD were significantly higher (2‐fold) than in healthy cats, but unfortunately the study groups were not age‐matched with a younger healthy group being compared to the CKD cats.^90^ The other study showed that uALB/Cr had poor diagnostic value when differentiating cats with azotemic CKD from healthy cats, but it tended to be higher in cats with azotemic CKD.^33^ In a more recent study, uALB/Cr was shown to have high sensitivity and specificity for classifying the severity of proteinuria in cats with various diseases. This ratio could distinguish healthy from diseased cats, but it could not differentiate between CKD cats and cats with non‐renal diseases.^86^ This result was supported by several studies in cats that detected microalbuminuria in a wide variety of non‐renal diseases, suggesting that albuminuria is not specific for renal diseases in cats.^23^, ^87^, ^88^, ^89^, ^110^ Moreover, it has been suggested that the clinical importance of the uALB/Cr ratio has not yet surpassed that of the UPC, which is routinely available in commercial laboratories.^9^, ^18^ Therefore, the clinical implication of albuminuria in cats with renal diseases is unclear.

#### 4.1.2 Transferrin

Transferrin is an ion‐binding glycoprotein synthesized in the liver and other tissues. Because its MW (77 kDa) is similar to that of ALB, transferrinuria is expected to be present when there is glomerular damage. In a study in humans, transferrinuria showed potential as a sensitive indicator of early diabetic nephropathy and a predictor of microalbuminuria development.^112^ However, urinary transferrin concentration was not associated with the progression of renal disease in another study in humans.^113^

A single study has investigated urinary transferrin concentrations in cats using sodium dodecyl sulfate‐polyacrylamide gel electrophoresis (SDS‐PAGE). This study showed that cats with IRIS stage 1 CKD had higher urinary transferrin concentrations than did healthy cats. With a cutoff of 0.93 mg/dL, urinary transferrin concentration was more sensitive and specific than plasma creatinine concentration for the detection of early‐stage CKD.^90^ Because of limited data availability, it is currently unknown whether increased urinary transferrin concentration reflects primarily tubulointerstitial nephritis or primary glomerular disease, taking into account that tubulointerstitial nephritis is the most common lesion in CKD cats.

### 4.2 Biomarkers of tubular injury or dysfunction

#### 4.2.1 Neutrophil gelatinase‐associated lipocalin

Neutrophil gelatinase‐associated lipocalin (NGAL) is produced by neutrophils and epithelial cells in various tissues, including renal tubular cells.^114^ It contributes to the innate immune response as a bacteriostatic agent and also aids in limiting damage after renal tubular injury.^115^ Neutrophil gelatinase‐associated lipocalin is expressed in response to infection, inflammation, and neoplasia.^116^, ^117^ Circulating NGAL passes freely through the glomerular barrier and is reabsorbed in the proximal tubules.^115^, ^118^ Renal injury impairs tubular reabsorption of NGAL and NGAL is released from the damaged renal tubular cells. Consequently, an increased NGAL concentration in the serum or urine may reflect tubular injury.^119^, ^120^, ^121^ In humans, both sNGAL and uNGAL concentrations appear to be promising biomarkers for the detection and prediction of AKI as well as CKD.^122^, ^123^, ^124^ In dogs, sNGAL is prognostic for survival in dogs with CKD and the urinary NGAL : creatinine ratio (uNGAL/Cr) has been suggested to be a useful biomarker for the detection of early‐stage CKD and predicting CKD progression in dogs.^125^, ^126^, ^127^, ^128^

An in‐house sandwich ELISA using anti‐canine NGAL antibodies has been established for measuring pNGAL and uNGAL concentrations in cats, with acceptable precision and repeatability.^94^ Recently, a commercial ELISA using anti‐human NGAL antibodies has been validated for sNGAL and uNGAL measurements in cats.^91^ In humans, uNGAL remains stable for at least 96 hours at 4°C and at least 6 months at −80°C, slowly decreasing with storage at −80°C over 5 to 8 years.^129^ Urinary NGAL concentrations in both humans and dogs decrease only slightly after 3 freeze‐thaw cycles.^6^, ^130^

The effect of biological status on NGAL concentrations has not been reported in cats, except for age, which does not influence uNGAL/Cr.^94^ Circulating NGAL concentrations do not affect uNGAL concentrations in cats.^91^, ^94^ Urinary tract infections (UTI) and pyuria increase uNGAL concentrations in both dogs and cats.^95^, ^131^ Therefore, lower urinary tract disease or UTI should be ruled out before interpreting uNGAL concentration as a biomarker of renal disease in these species. Again, with respect to specificity, increased uNGAL/Cr ratios also have been found in cats with a variety of non‐renal diseases.^94^, ^95^ One study compared uNGAL/Cr among CKD cats, cats with AKI, and healthy cats. Higher uNGAL/Cr ratios were found in cats with CKD and AKI compared to healthy cats. The highest uNGAL/Cr ratios were detected in the AKI group, which probably was related to the more severe azotemia in that group compared to the CKD group.^95^

To date, studies that evaluate NGAL as early renal biomarker in CKD cats are limited, and conflicting results have been found. In a study evaluating uNGAL concentration in 80 cats with azotemic CKD and 18 healthy cats, uNGAL/Cr ratio was significantly increased in the former group, particularly in cats with IRIS stages 3 and 4 CKD.^94^ In contrast, a recent study evaluating 9 CKD cats and 44 healthy cats reported that uNGAL/Cr was not different between the groups. The reason for the discordant findings between these studies is unclear, but might be because of the different assays used.^91^ The former study also assessed the prognostic utility of uNGAL concentrations in CKD cats and found higher baseline uNGAL/Cr concentrations in the group with progression compared to the group without progression.^94^ Unfortunately, this finding must be interpreted cautiously because of important study limitations such as the different severity of azotemia between both groups at baseline and the short duration of follow‐up (30 days).^94^ Additional studies are needed to determine whether uNGAL/Cr indeed can be used to predict progression of CKD in cats in clinical practice.

Similar to humans and dogs, NGAL in cats has 3 different molecular forms including monomeric (25 kDa), dimeric (45 kDa), and NGAL/matrix metalloproteinase‐9 (MMP9) complexes (135 kDa). The monomeric form is mainly detected in azotemic cats with AKI and late‐stage CKD (ie, stages 3 and 4).^95^ Its presence may reflect advanced tubular injury or impairment, which may be useful as a prognostic factor in cats with AKI or CKD. The monomer was also the predominant form of uNGAL detected in a group of non‐pyuric cats with non‐renal diseases.^95^ The monomeric form is valuable primarily to investigate the diagnostic potential of NGAL in AKI and as biomarker for progression of CKD. The dimeric form is predominantly present in cats with UTI and pyuria, emphasizing that UTI should be ruled out when interpreting uNGAL concentrations in cats.^95^ Interestingly, the NGAL/MMP9 complex is commonly found in the urine of cats independent of their health status, which is a notable difference compared to humans and dogs.^95^, ^132^, ^133^, ^134^ This finding may infer that healthy cats physiologically express NGAL/MMP9 complex, or unknown pathological conditions are present in these cats.

Based on results of the available studies, uNGAL may relate more to the severity of azotemia or renal impairment rather than the type and progression of kidney disease. Also, the limitation of using non‐feline specific assays for NGAL measurements in cats must be considered.

#### 4.2.2 Liver‐type fatty acid‐binding protein

Liver‐type fatty acid‐binding protein (L‐FABP) is a 14‐kDa protein located in the cytoplasm of hepatocytes and proximal tubular epithelial cells.^135^, ^136^ It also is expressed in the intestine, lung, and pancreas.^137^ Circulating L‐FABP normally passes through the glomerular barrier and is reabsorbed in the proximal tubules. In the kidneys, L‐FABP plays a major role in fatty acid homeostasis, resulting in fatty acid catabolism that provides energy to proximal tubular cells.^136^, ^138^ Moreover, it has protective effects on the proximal tubules by transporting harmful lipid peroxidation products from the cytoplasm into the tubular lumen.^136^, ^139^ Ischemic injury and oxidative stress of the proximal tubules result in L‐FABP excretion into the urine in small laboratory animals and humans.^135^, ^140^ In the clinical setting, uL‐FABP/Cr ratio is a biomarker of early tubular damage that can reflect the severity of tubulointerstitial injury, detect AKI, and predict CKD progression in humans.^141^, ^142^, ^143^, ^144^, ^145^, ^146^, ^147^ Furthermore, L‐FABP has been shown to be renoprotective in small laboratory animals.^148^, ^149^

A commercial feline L‐FABP ELISA based on cross‐reactivity with anti‐human L‐FABP has been used in research on cats and recently has been validated for feline serum and urine.^91^, ^92^, ^93^ Human uL‐FABP was stable at 4°C for 48 hours and at −70°C for at least 18 months.^150^, ^151^ The stability of L‐FABP in samples from cats has not been reported.

In humans, other non‐renal factors are known to influence uL‐FABP concentrations.^141^, ^143^, ^152^, ^153^, ^154^, ^155^, ^156^ In non‐diabetic human patients, age, sex, and serum cholesterol concentration are not associated with uL‐FABP/Cr, whereas this biomarker significantly increases in anemic patients.^154^ Proteinuria, albuminuria, and hematuria are strongly associated with uL‐FABP/Cr, whereas pyuria has very little impact.^141^, ^143^, ^153^, ^156^ Liver disease significantly influences sL‐FABP concentrations but not uL‐FABP/Cr. Also, sL‐FABP concentration did not correlate with uL‐FABP/Cr in humans with CKD and liver disease.^152^ Nevertheless, uL‐FABP/Cr was correlated with various liver enzymes in critically ill humans.^155^ In cats, the current information about the effect of non‐renal factors on L‐FABP is limited. It is only known that uL‐FABP/Cr is significantly increased in hyperthyroid cats, and resolved after euthyroidism was established.^91^ It remains unknown whether uL‐FABP concentration can predict post‐treatment azotemia in hyperthyroid cats because of a low number of post‐treatment azotemic cats in this study.^91^

Currently, only 3 studies have evaluated L‐FABP in cats.^91^, ^92^, ^93^ It was detected in the cytoplasm of proximal tubular cells in healthy feline kidneys using immunohistochemistry with antibodies targeting human L‐FABP, and it was expressed in the tubular lumen in response to ischemic‐reperfusion injury.^92^ Most healthy control cats had uL‐FABP/Cr ratios below the limit of detection,^91^, ^92^ whereas this ratio increased in an AKI model in cats immediately after inducing ischemic‐reperfusion injury.^92^ Although uL‐FABP/Cr was significantly increased in CKD cats compared to healthy cats, an overlap between groups was noted.^91^, ^93^ Urinary L‐FABP/Cr was high (>10 μg/g) in approximately 50% of cats with azotemic CKD and in only 7% of non‐azotemic cats.^93^ Also, the cutoff of 0.97 μg/g was able to distinguish azotemic CKD cats from healthy cats with 100% sensitivity and 93% specificity.^91^ Because uL‐FABP concentration appears to be a sensitive biomarker for tubular stress or injury, cats having increased uL‐FABP/Cr are thought to have active tubular stress or injury regardless of the presence of renal structural and functional changes. Higher uL‐FABP/Cr may indicate more advanced tubular injury or loss. Increased uL‐FABP/Cr in non‐azotemic cats might suggest increased odds for development of AKI or CKD in the future, but this possibility should be confirmed by additional studies. In particular, studies evaluating non‐renal factors affecting uL‐FABP/Cr in cats and prospective, longitudinal studies of non‐azotemic cats with increased uL‐FABP/Cr are lacking. Also, the reference interval for uL‐FABP/Cr needs to be established in a larger population of healthy cats.

#### 4.2.3 Kidney injury molecule‐1

Kidney injury molecule‐1 (KIM‐1) is a type‐1 membrane glycoprotein.^157^ It is a cell surface receptor in epithelial, lymphoid, and myeloid cells that scavenges oxidized circulating low‐density lipoproteins and membrane‐associated phosphatidylserine.^158^ No KIM‐1 protein was detectable in the urine of healthy humans.^159^ With renal tubular damage, KIM‐1 is upregulated in proximal tubular cells.^160^, ^161^ Several studies in humans have reported an association between uKIM‐1 and both AKI severity and rapid CKD progression.^159^, ^160^, ^161^, ^162^, ^163^, ^164^ In dogs, uKIM‐1 has been suggested as a promising biomarker for the detection of both naturally occurring AKI and CKD.^165^, ^166^

Feline KIM‐1 has been detected in urine using the commercially available anti‐rat KIM‐1 lateral flow immunoassay.^167^ More recently, the same research group also developed a specific lateral flow assay for the detection of feline uKIM‐1.^96^ In mice, uKIM‐1 is stable for 5 days at room temperature and at least for 1 year at −80°C, and multiple freeze–thaw cycles do not significantly alter uKIM‐1 concentrations.^168^ The effects of storage time and temperature on uKIM‐1 concentrations in cat samples remain unknown.

Regarding the influence of non‐renal factors, age may positively affect uKIM‐1 concentrations in humans,^169^, ^170^ as might ethnic origin, with those of white European ancestry having higher uKIM‐1 concentrations than African Americans.^171^ A difference in uKIM‐1 concentrations between the sexes in humans is debatable.^169^, ^171^ In humans, uKIM‐1 concentrations correlate with proteinuria severity but are unaffected by the presence of erythrocytes or leukocytes.^172^ No studies have evaluated the influence of non‐renal factors on KIM‐1 concentrations in cats.

One research group performed 3 studies on uKIM‐1 as a biomarker for the detection of kidney disease in cats. The first investigated the presence of feline uKIM‐1 using the urine anti‐rat immunoassay. Kidney injury molecule‐1 was present in the urine of cats with critical conditions associated with AKI but was undetectable in all healthy cats. By immunohistochemistry, KIM‐1 localized specifically to proximal tubules in the outer medulla and in luminal cell debris in feline kidneys. Moreover, positive uKIM‐1 immunoassay results were consistent with KIM‐1 immunohistochemical staining and histopathological findings in kidney tissue sections. Nevertheless, 3 of 6 CKD cats had negative immunohistochemical staining results. The authors suggested that this apparent contradiction might have been associated with fibrotic changes and loss of the specific localization of KIM‐1 in renal tissue sections because of small biopsy sample size.^167^ In their second study, the authors found that healthy cats had no positive KIM‐1 immunohistochemical staining, whereas cats with experimental and naturally occurring AKI showed increased expression of KIM‐1 in the proximal tubules.^173^ Their recent third study showed an overlap between uKIM‐1 concentrations in cats with suspected AKI and healthy cats using an in‐house feline‐specific KIM‐1 immunoassay. The concentrations in cats with suspected AKI were highly variable, whereas healthy cats had low and non‐variable uKIM‐1 concentrations. Some cats (31%) with conditions associated with AKI had a substantial but transient increase in uKIM‐1. Only 1 CKD cat expressed uKIM‐1 at lower levels than healthy cats.^96^ Overall, uKIM‐1 is highly expressed after early severe tubular injury but further tubular loss may result in negative results. Therefore, kinetic monitoring of this biomarker using serial sample analysis might be important in practice. Finally, KIM‐1 seems to have greater potential in the AKI setting compared to the CKD setting in cats, but further evaluation of uKIM‐1 in CKD cats is warranted.

#### 4.2.4 Vascular endothelial growth factor

Vascular endothelial growth factor (VEGF), also known as vascular permeability factor, is a signaling protein affecting angiogenesis.^174^ It is expressed in renal proximal tubular cells in response to hypoxia.^175^ In normal human kidneys, VEGF is constantly produced to maintain the glomerular and peritubular vasculature.^176^ Urinary VEGF expression is downregulated in progressive CKD in humans.^176^, ^177^, ^178^, ^179^ Thus, uVEGF is a potential prognostic indicator for kidney function loss.

Very few studies on VEGF in CKD have been performed in cats.^97^, ^98^, ^180^ The commercially available human VEGF ELISA cross‐reacts with its feline homolog and has been validated for the detection of feline uVEGF.^180^ In CKD cats, uVEGF concentrations were significantly lower than in healthy cats, with considerable overlap between groups. The association between uVEGF/Cr and sCr was not significant in these CKD patients.^97^ Hyperthyroid cats also had significantly higher uVEGF/Cr ratios than healthy cats. Upon treatment, uVEGF significantly decreased but it remained significantly higher than in healthy cats. Moreover, hyperthyroid cats with post‐treatment renal azotemia had lower uVEGF/Cr both pre‐ and post‐treatment compared to those that remained non‐azotemic after treatment. Additionally, uVEGF/Cr was negatively correlated with sCr but positively correlated with plasma total thyroxine concentrations, plasma renin activity, and UPC in hyperthyroid cats.^98^ Consequently, increased uVEGF/Cr in hyperthyroid cats may not reflect renal dysfunction, whereas abnormal uVEGF/Cr may indicate decreased renal mass or function in hyperthyroid cats.^98^ Overall, the higher uVEGF/Cr in non‐azotemic compared to CKD cats may indicate a renoprotective effect of VEGF in healthy kidneys, and a low uVEGF/Cr in healthy cats may indicate early CKD.^97^ Based on these findings, the exact role of VEGF in the feline kidney remains unclear and additional studies are required to determine the diagnostic and prognostic value of uVEGF in early CKD in cats.

#### 4.2.5 Urinary cystatin C

Serum CysC concentration was discussed in Section 2.1.1 as a GFR biomarker. However, tubular renal damage decreases tubular reabsorption of CysC, resulting in increased excretion.^181^ Therefore, uCysC concentration has been suggested as a promising biomarker for the assessment of renal tubular function in humans and dogs. In humans, uCysC/Cr was found to be useful as a tubular damage biomarker in patients with CKD.^181^ In dogs, uCysC/Cr can be used to distinguish dogs with renal disease from dogs with non‐renal disease.^182^

The anti‐human‐based PETIA and PENIA kits have been validated for uCysC in cats, although the former has poor repeatability in the lower concentration range.^31^, ^32^, ^33^ In humans, uCysC is stable at both −20 and 4°C for 7 days and even at 20°C for 48 hours.^183^ Urinary CysC concentration is increased in humans with proteinuria, but the effect of proteinuria on uCysC concentration has not been studied in cats.^184^ Urinary CysC/Cr ratios were significantly increased in hyperthyroid cats but not in cats with diabetic mellitus.^24^, ^185^

One study found that uCysC/Cr was significantly higher in CKD cats compared to healthy cats. Nevertheless, 34% of CKD cats had undetectable uCysC concentrations vs 88% of healthy cats.^32^ A more recent study observed that uCysC/Cr was significantly lower in CKD cats compared to healthy cats.^33^ These different findings among studies might be a consequence of different CKD stages of the cats included or different analytical methods used. In the former study, CKD cats had more advanced disease and higher median UPC than those in the latter study.^32^, ^33^ Moreover, the usefulness of uCysC/Cr as a screening test to distinguish azotemic CKD cats from healthy cats was not better than USG.^33^ These poor clinical correlations may be a result of the disadvantage of the current assays such as sCysC. Therefore, with the currently available assays, uCysC does not seem to be a reliable biomarker for detection of early CKD in cats.

#### 4.2.6 Heat shock protein‐72

Heat shock protein‐72 (HSP‐72) is a stress‐induced HSP isoform, which plays a role in protecting cell and protein structure upon cellular insult and stress conditions.^186^, ^187^ It is synthesized by a variety of cells including renal tubular cells.^188^ In children undergoing renal transplantation, urinary HSP72 excretion was substantially increased during the early post‐transplant period.^189^ In dogs, uHSP72/Cr was shown to be a potential renal biomarker for AKI.^190^

A commercial HSP72 ELISA kit for cats has been validated by its manufacturer and used in a recent study in cats.^99^ Urinary HSP72 concentrations were higher in cats with AKI, CKD, urethral obstruction, and acute‐on‐chronic kidney disease. Furthermore, uHSP72/Cr could distinguish cats with AKI from healthy cats with 94% sensitivity and 70% specificity using a cutoff of 0.54 ng/mg. However, few cats in the early stages of disease were included, as only 2 of 16 AKI cats had AKI grade 1, and 3 of 15 CKD cats were IRIS stage 1. Also, an overlap was noticed in uHSP72/Cr between CKD and healthy cats. Thus, the clinical value of HSP72 to detect early AKI and CKD in cats remains uncertain and additional investigations in a larger group of cats are warranted. Moreover, non‐renal factors affecting HSP72 in cats have not been studied.

#### 4.2.7 F_2_ ‐isoprostanes

F_2_‐isoprostanes are prostaglandin‐like metabolites synthesized locally in the kidney through peroxidation of the common precursor arachidonic acid by glomerular endothelial and mesangial cells.^191^ These lipids are reliable indicators of oxidative injury and lipid peroxidation.^192^ Oxidative stress can induce tubulointerstitial injury and fibrosis.^193^, ^194^ Plasma F_2_‐isoprostane concentrations were increased in human CKD patients,^195^, ^196^, ^197^, ^198^ whereas uF_2_‐isoprostane concentrations in humans with CKD were lower than in healthy humans.^199^ Another study demonstrated that uF_2_‐isoprostane concentrations correlated positively with eGFR in elderly men.^200^

Affinity column purification followed by a competitive ELISA has been used to quantify uF_2_‐isoprostane concentrations in cats. This method weakly correlates with gas chromatography/mass spectrometry, which remains the gold standard for isoprostane measurement.^201^ Thus, the validity of the data assessed using the ELISA method remains doubtful. Hypertension and proteinuria were not associated with uF_2_‐isoprostane concentrations in cats.^100^ Urinary F_2_‐isoprostane concentrations were, however, increased in hyperthyroid cats compared to healthy cats and normalized after treatment.^101^

Although cats with stage 1 CKD had higher uF_2_‐isoprostane concentrations than healthy cats, this difference was not significant. The authors suggested that this finding might be a consequence of insufficient statistical power when comparing CKD stage 1 cats and healthy cats. With advancing CKD stage (stages 3 and 4), uF_2_‐isoprostane concentrations decreased significantly compared to concentrations in stage 1 CKD and healthy cats. No correlation was observed between uF_2_‐isoprostane and sCr concentrations.^100^ Despite several limitations, these findings suggest that oxidative stress may be transiently active in cats with stage 1 CKD, and may be a diagnostic target for early detection of CKD. Because of limited information and several limitations, further study to demonstrate the potential of uF2‐isoprostane concentration as an early renal biomarker in CKD cats is less compelling compared to other renal biomarkers.

#### 4.2.8 Others

Novel biomarkers in veterinary medicine are cystatin B (CysB) and urinary clusterin. Cystatin B is a low‐molecular weight protein (11 kDA) that functions to inhibit members of the cysteine proteases (family 1). Cystatin B is mainly an intracellular protein with limited concentration in the circulation.^27^ Clusterin is a glycoprotein (70‐80 kDA) produced in various tissues during several physiological and pathological processes.^27^, ^202^, ^203^ Normally, clusterin is found in very low concentration in urine. Urinary clusterin (uClust) concentration is increased during tubular injury in dogs.^204^, ^205^ In people, uClust is part of renal biomarker panels for drug development and toxicity.^27^ For both markers, concentrations can be determined in dogs and cats using sandwich immunoassays.^27^ To accurately detect active tubular injury in samples from dogs and cats, the kidney‐specific isoform of clusterin is measured.^27^

Studies have focused on the diagnostic potential of these markers in dogs and cats with AKI. Urinary CysB is increased in dogs with AKI and is shown not to be affected by UTI.^27^, ^206^ Similarly, an abstract reported that cats with AKI had significantly higher uCysB concentrations than healthy cats and that uCysB can predict AKI with 90% sensitivity and 92% specificity.^207^ In dogs, uClust concentration is increased in AKI.^27^, ^206^, ^208^

The only study evaluating uCysB and uClust concentrations in CKD cats did not find a difference in the concentrations of those and other renal biomarkers between cats treated with low‐dose meloxicam and cats treated with placebo.^209^ Current scientific information in cats is too limited to draw conclusions on the clinical utility of CysB and clusterin to diagnose CKD in cats.

### 4.3 Biomarkers of renal fibrosis

The most common histopathological diagnosis in CKD in cats is interstitial inflammation and fibrosis.^210^ Among the histopathological findings in CKD in cats, interstitial fibrosis is most closely associated with azotemia severity, hyperphosphatemia, anemia, and proteinuria.^8^ These findings suggest that renal fibrosis and its mediators play crucial roles in CKD pathogenesis. Renal biomarkers indicating renal fibrosis therefore may be of benefit for the early detection and treatment of CKD in cats.

#### 4.3.1 Transforming growth factor‐β1

Transforming growth factor‐β1 (TGF‐β1) is a multifunctional cytokine that potentially plays a role as a profibrotic mediator activating tissue fibrosis.^211^ It is produced by parenchymal and inflammatory cells in several organs and especially the kidneys.^212^, ^213^, ^214^, ^215^, ^216^ In the kidneys, TGF‐β1 initially is released in an inactive form in response to various renal insults such as oxidative stress, hypoxia, proteinuria, and products of the renin‐angiotensin‐aldosterone system.^217^, ^218^, ^219^, ^220^ Inactive TGF‐β1 then must be altered to its active form by proteolytic cleavage before promoting renal fibrosis.^221^, ^222^ The profibrotic effect of TGF‐β1 consists of myofibroblast formation, which promotes extracellular matrix production, decreased extracellular matrix degradation, and tubular injury and cellular apoptosis.^84^, ^223^, ^224^, ^225^, ^226^ It also has an important role in tissue homeostasis, recruiting stem cells in tissue reparative processes.^227^ Increased urinary TGF‐β1 (uTGF‐β1) expression is found in rodents and humans with kidney disease related to interstitial fibrosis.^228^, ^229^ Overall, studies in humans have shown significant upregulation of uTGF‐β1 expression in patients with glomerular diseases.^228^, ^229^, ^230^ Urinary TGF‐β1 concentrations were significantly correlated with the severity of interstitial fibrosis in renal biopsy samples but did not correlate with sCr and GFR.^228^, ^230^

In CKD cats, a commercial multispecies total TGF‐β1 ELISA has been used to measure total urinary TGF‐β1 concentrations in cats. However, no validation data have been reported for feline urine using this ELISA.^97^, ^102^ A commercial active TGF‐β1 (aTGF‐β1) ELISA for humans, which detected an active form of TGF‐β1 in urine, later was validated for cats with acceptable precision and reproducibility.^103^

In rodent models, renal interstitial fibrosis was associated with aging, resulting in increased TGF‐β1 expression in the kidneys.^231^ However, it is unknown whether this phenomenon occurs in cats. Proteinuria may influence uaTGF‐β1 concentrations, because significant correlation was found between log UPC and uaTGF‐β1 : creatinine ratio (uaTGF‐β1/Cr).^103^

Few studies have evaluated TGF‐β1 for assessing renal fibrosis in CKD cats. Cats with CKD had higher urinary total TGF‐β1 concentrations than did healthy cats, whereas serum TGF‐β1 concentration was not different between groups.^97^, ^102^ Urinary total TGF‐β1/Cr also was positively correlated with sCr concentrations.^97^ In contrast, another study was unable to measure total TGF‐β1 concentrations in feline urine using the same ELISA.^103^ Moreover, baseline uaTGF‐β1/Cr ratios were not different between CKD cats and healthy cats, suggesting that CKD cats might not express more uaTGF‐β1 than healthy cats.^103^ However, non‐azotemic cats that later developed renal azotemia had a 2.6‐fold increase in uaTGF‐β1/Cr from baseline approximately 6 months before azotemia was detected. These results suggest that rather than sampling at a single time point, serial measurements in non‐azotemic geriatric cats of uaTGF‐β1/Cr may be useful to predict azotemic CKD later in life.^103^ Additionally, uaTGF‐β1/Cr moderately correlated with the severity of renal interstitial fibrosis in this histopathologic study.^103^ In vitro, TGF‐β1 induced the expression of genes associated with the pathogenesis of renal fibrosis in the proximal tubular epithelial cells of cats.^232^ These findings support that TGF‐β1 might be involved in the pathogenesis of CKD in cats by inducing pro‐fibrotic factors related to renal fibrosis, and uaTGF‐β1 expression may reflect renal fibrosis severity in cats. Overall, TGF‐β1 may be promising to identify the likelihood of development of azotemic CKD in non‐azotemic geriatric cats.

#### 4.3.2 Procollagen type III amino‐terminal propeptide

Procollagen type III amino‐terminal propeptide (PIIINP), a LMW (44 kDa) peptide, is cleaved and released from collagen into extracellular fluids including blood during the synthesis and deposition of type III collagen. Circulating PIIINPs can pass through glomeruli and are reabsorbed by proximal tubular cells.^233^ In the absence of renal damage, only a small amount of PIIINP can be detected in human urine. However, urinary PIIINP expression is increased in renal fibrosis.^234^ This molecule has been studied for its utility as a novel biomarker of fibrosis in various organs such as lung, heart, liver, and kidney. Many studies in humans and dogs have shown that plasma PIIINP concentrations are increased in patients with diseases characterized by fibrosis.^235^, ^236^, ^237^, ^238^, ^239^ The urinary PIIINP : creatinine ratio (uPIIINP/Cr) is negatively correlated with eGFR and positively associated with the progression of CKD and severity of renal fibrosis in humans.^240^, ^241^, ^242^, ^243^

A single study has evaluated PIIINP in small groups of cats using a sandwich ELISA. No validation data of the ELISA was reported. The study showed significantly increased uPIIINP/Cr in cats with azotemic CKD compared with healthy cats. The uPIIINP/Cr ratio also was highly correlated with renal parenchyma stiffness as determined by ultrasonic shear‐wave ultrasonography.^104^ Currently, information of PIIINP in cats remains scarce. Additional studies using validated assays are required to establish whether PIIINP is a promising biomarker for CKD in cats.

## 5 CONCLUSIONS

Several biomarkers have shown value or are promising in the diagnostic evaluation of CKD in cats. Rather than detecting decreased GFR, the true power of these biomarkers lies in their other roles, especially the ability to identify acute kidney stress or injury or active pathological changes. Several renal biomarkers show potential for early detection or for determining progression in CKD in cats. Taking these characteristics into account, longitudinal monitoring of these biomarkers may be superior to individual measurements for the determination of the renal status of individual cats.

Before any of these renal biomarkers can be used in clinical practice, steps of validation are needed. First, for the concentration of a biomarker to be valuable in samples from cats, thorough analytical validation of available assays is needed. Second, biological validation is needed by establishing reference intervals in healthy cats and by assessing the potential influence of biological factors. Third, it is important to evaluate whether the biomarkers can reliably differentiate the renal status of the cats (ie, non‐azotemic healthy vs non‐azotemic early CKD vs azotemic CKD), which represents clinical validation. Choosing the decision threshold of certain biomarkers also is required in this step. The true diagnostic value of biomarkers will depend on the combined analytical, biological, and clinical validation. Also, it is important to evaluate whether the additional information provided by the biomarkers results in additional benefits for the diagnosis and clinical‐decision making in cats with CKD. Unfortunately, most of the biomarkers discussed here are not yet fully validated. Important limitations to assess the clinical utility of renal biomarkers in living cats are the difficulty to correlate biomarker concentrations with pathological changes within the kidney and the fact that most of the current data result from cross‐sectional studies. Additional investigations still are needed before determining the usefulness of these biomarkers in clinical practice, preferably by performing longitudinal studies. Also, for daily use in the clinic, easily accessible, reliable, and inexpensive assays should become available for the most promising biomarkers.

## CONFLICT OF INTEREST DECLARATION

Authors declare no conflict of interest.

## OFF‐LABEL ANTIMICROBIAL DECLARATION

Authors declare no off‐label use of antimicrobials.

## INSTITUTIONAL ANIMAL CARE AND USE COMMITTEE (IACUC) OR OTHER APPROVAL DECLARATION

Authors declare no IACUC or other approval was needed.

## HUMAN ETHICS APPROVAL DECLARATION

Authors declare human ethics approval was not needed for this study.
